# Global research trends in MERS-CoV: A comprehensive bibliometric analysis from 2012 to 2021

**DOI:** 10.3389/fpubh.2022.933333

**Published:** 2022-08-04

**Authors:** Tauseef Ahmad

**Affiliations:** ^1^Vanke School of Public Health, Tsinghua University, Beijing, China; ^2^Department of Epidemiology and Health Statistics, School of Public Health, Southeast University, Nanjing, China

**Keywords:** MERS-CoV, bibliometric analysis, HistCite^TM^, VOSviewer software, WoSCC database, COVID-19

## Abstract

**Background:**

The Middle East respiratory syndrome coronavirus (MERS-CoV) was first reported in Saudi Arabia in 2012. So far, the cases of MERS-CoV have been reported in 27 countries. The virus causes severe health complications, resulting high mortality.

**Aim:**

The current study aimed to evaluate the global research trends and key bibliometric indices in MERS-CoV research from 2012 to 2021.

**Methods:**

A retrospective bibliometric and visualized study was conducted. The Science Citation Index Expanded Edition of Web of Science Core Collection database was utilized to retrieve published scientific literature on MERS-CoV. The retrieved publications were assessed for a number of bibliometric attributes. The data were imported into HistCite^TM^ and VOSviewer software to calculate the citations count and perform the visualization mapping, respectively. In addition, countries or regions collaboration, keywords analysis, and trend topics in MERS-CoV were assessed using the Bibliometrix: An R-tool.

**Results:**

A total of 1,587 publications, published in 499 journals, authored by 6,506 authors from 88 countries or regions were included in the final analysis. Majority of these publications were published as research article (*n* = 1,143). Globally, these publications received 70,143 citations. The most frequent year of publication was 2016 (*n* = 253), while the most cited year was 2014 (11,517 citations). The most prolific author was Memish ZA (*n* = 94), while the most published journal was Emerging Infectious Diseases (*n* = 80). The United States of America (USA) (*n* = 520) and Saudi Arabia (*n* = 432) were the most influential and largest contributors to the MERS-CoV publications. The extensively studied research area was infectious diseases. The most frequently used author keywords other than search keywords were Saudi Arabia, SARS-CoV-2, COVID-19, epidemiology, transmission, spike protein, vaccine, outbreak, camel, and pneumonia.

**Conclusion:**

This study provides an insight into MERS-CoV-related research for scientific community (researchers, academicians) to understand and expand the basic knowledge structure, potential collaborations, and research trend topics. This study can also be useful for policy makers. After the emergence of MERS-CoV, a significant increase in scientific production was observed in the next 4 years (2013–2016). In 2021, the trend topics in MERS-CoV-related research were COVID-19, clinical characteristics, and cytokine storm. Saudi Arabia had the strongest collaboration with the USA, while the USA had the highest collaboration with China.

## Introduction

The first case of Middle East respiratory syndrome coronavirus (MERS-CoV) was reported from Saudi Arabia in 2012 (1). A 60-year-old man was admitted to a private hospital in Jeddah on June 13, 2012, with 1-week history of cough, expectoration, fever, and shortness of breath. On June 14, 2012, the patient died due to a rapidly deteriorating clinical course (1–3). After 3 months, a new β coronavirus previously known as human coronavirus Erasmus Medical Center virus was detected (4). Later on, the “Coronavirus Study Group of the International Committee on Taxonomy of Viruses” renamed the virus as MERS-CoV (5).

Globally, a total of 2,574 laboratory confirmed cases, along with 886 deaths, have been reported to World Health Organization (WHO) as of March 11, 2021 (6). In addition, between January 1, 2021 and December 6, 2021, 14 laboratory confirmed cases of MERS-CoV were reported in Saudi Arabia (*n* = 13), and the United Arab Emirates (*n* = 1), including five deaths (7). So far, the MERS-CoV cases have been reported in 27 countries (7).

**Figure 1:**
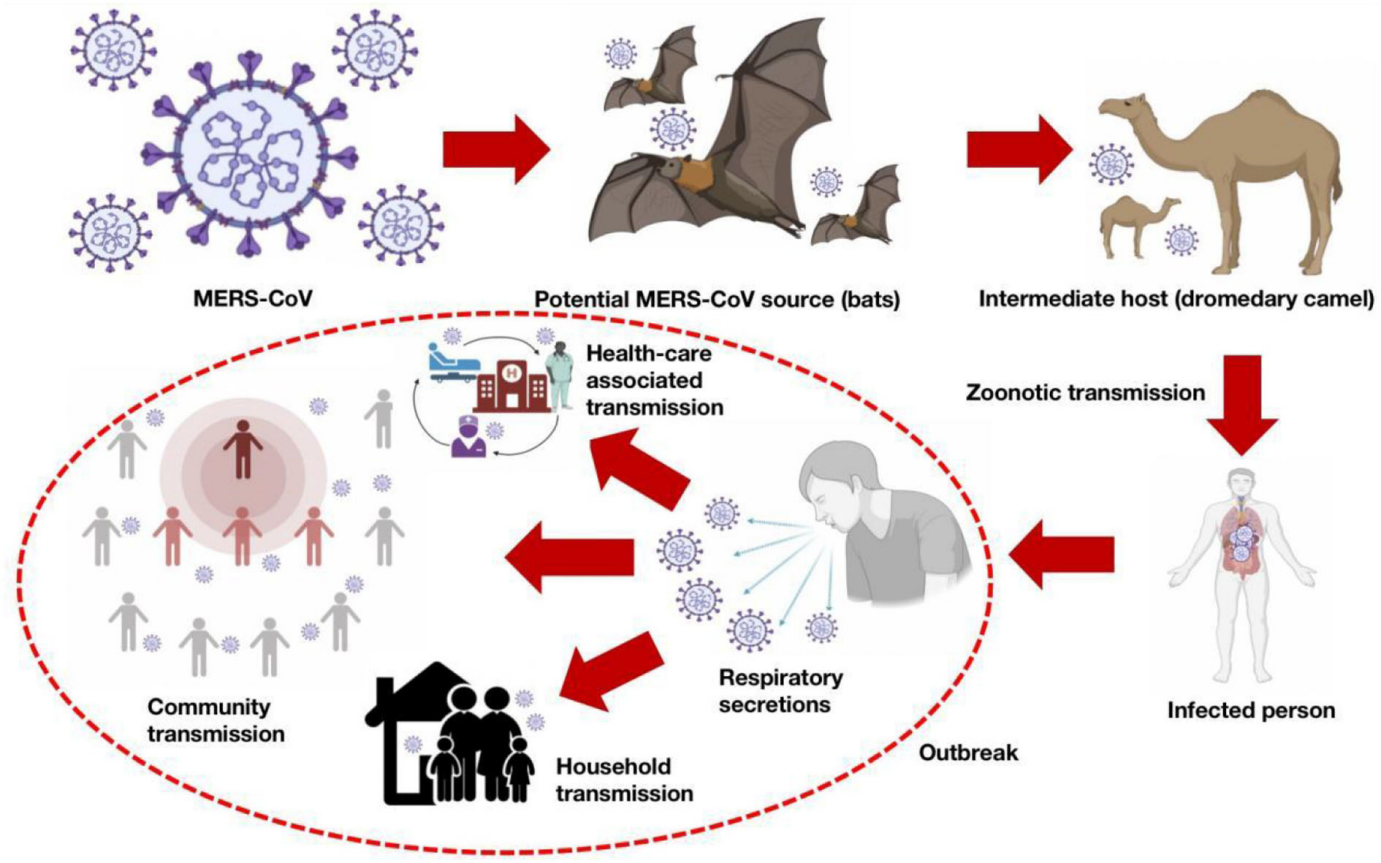
Potential routes of emergence and transmission of MERS-CoV.

Most importantly, infection with MERS-CoV can cause severe health complications, resulting in high mortality, specifically in patients with chronic lung diseases, diabetes, immunocompromised persons, and renal failure (6). MERS-CoV is zoonotic in nature, and bats are considered to be a potential reservoir, while a dromedary camel is an intermediate host. Sporadically, MERS-CoV is transmitted from a dromedary camel to humans, and, occasionally, *via* human-to-human contact (8–11). The potential routes of MERS-CoV emergence and transmission are presented in Figure 1. It is necessary to prevent or reduce zoonotic spillover events since 60–75% of human infectious diseases emerged from pathogens originally circulating in non-human animal species (12).

**Figure 2:**
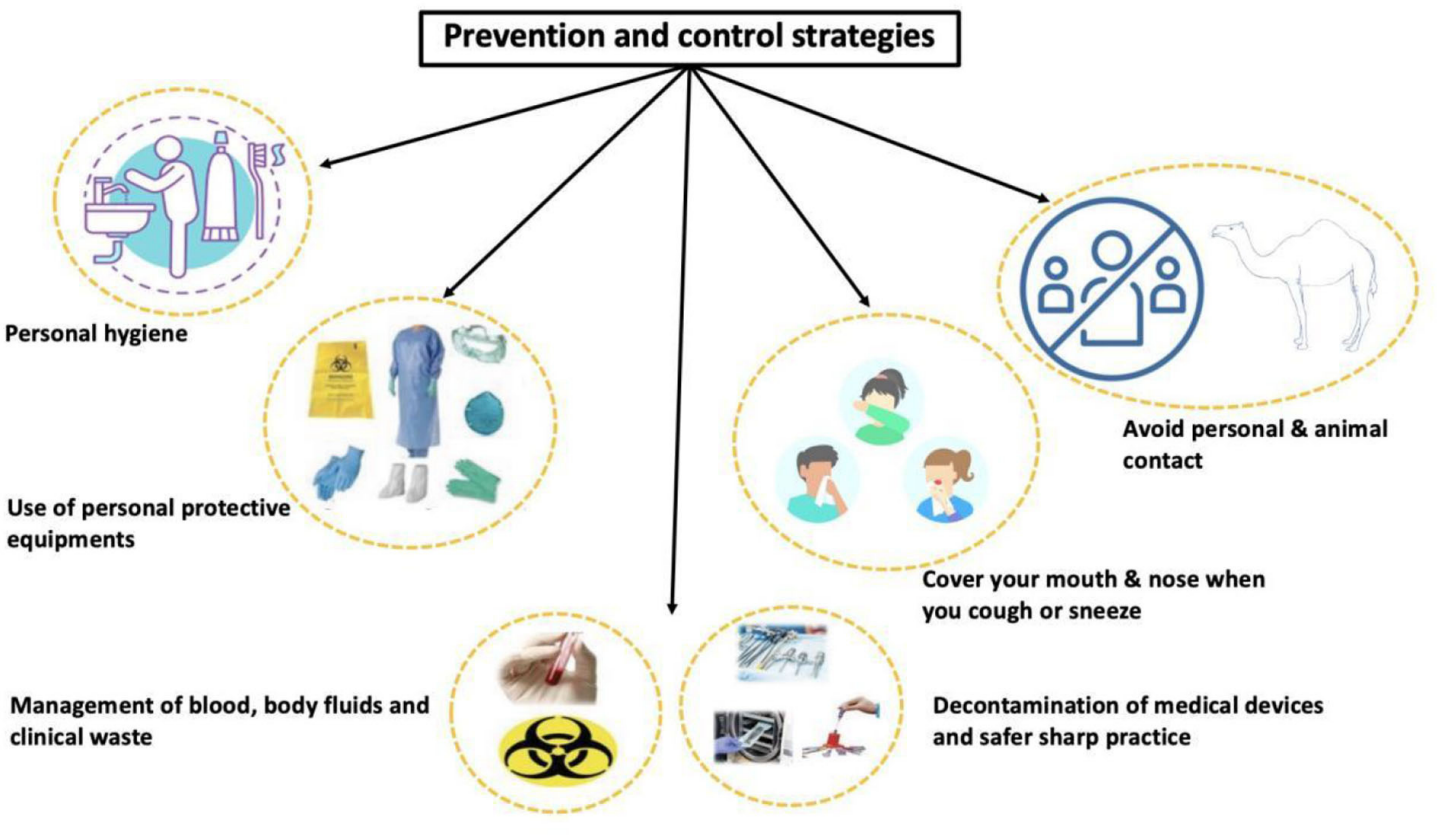
Prevention and control strategies for MERS-CoV.

In the light of available scientific literature, MERS-CoV possesses significant public health challenges (6). Moreover, good personal hygiene and medical practices should be followed to prevent the spread of MERS-CoV as shown in Figure 2.

Till to date, a number of bibliometric studies have been published on MERS-CoV (13–16).

Bibliometric methods are frequently used for quantitative and qualitative scholarly publications analyses and changes in research activity over time in a particular field or subject (17–19). Bibliometric analysis could be used as an objective criterion to assess and evaluate the research production by scientists, institutes, and countries (19, 20). Importantly, bibliometric analysis serves as a referral point of contact for policy makers and researchers, as well as a guide for future research direction (21). Therefore, the use of these methods has significantly increased over the last decade in medical and health sciences disciplines. However, there is no comprehensive updated bibliometric and visualization study available. Thus, the current study was conducted to determine the global research trends, achievements, and keystone bibliometric indices in MERS-CoV research during the past 10 years.

## Methods

### Study design

A retrospective bibliometric and visualized study was conducted.

### Data source and search operations

**Figure 3:**
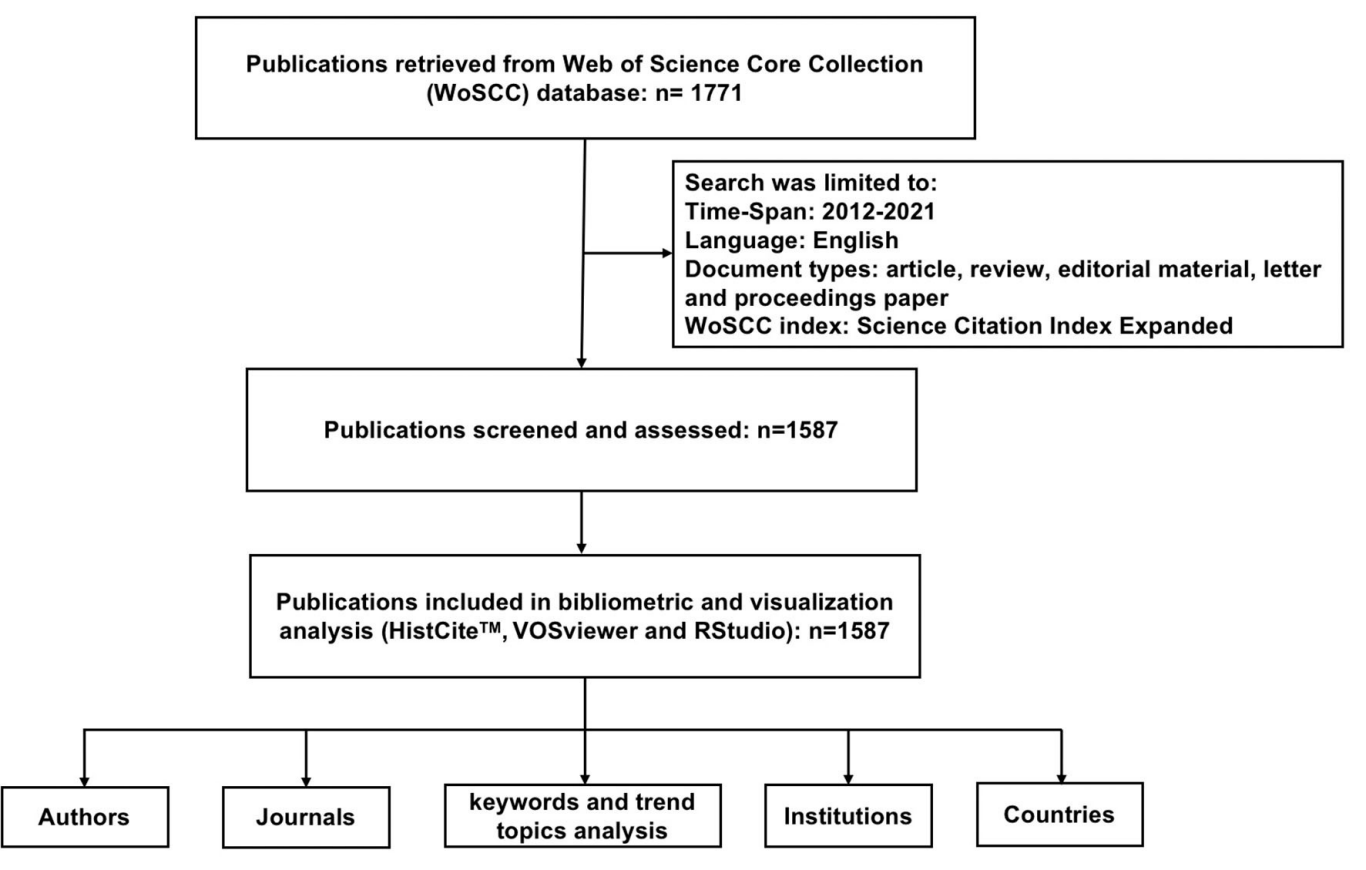
A publications selection flow chart included in the final analysis.

On January 1, 2022, the Science Citation Index Expanded (SCI-Expanded) Edition of Web of Science Core Collection (WoSCC) database was searched for the relevant scientific literature on MERS-CoV. The following searching keywords were utilized in the title field, applying the Boolean search method: “Middle East respiratory syndrome” or “Middle East respiratory syndrome coronavirus” or “MERS-CoV” or “human coronavirus Erasmus Medical Center virus” or “novel coronavirus.” The search was limited to publishing language (English), document types (article, review, editorial material, letter, and proceedings paper), and publications year (2012–2021). However, the search with a keyword “novel coronavirus” was limited from 2012 to 2019 to avoid the scientific publications on the recently emerged coronavirus (SARS-CoV-2). A total of 41 publications were retrieved with a keyword “novel coronavirus,” while the other keywords retrieved a total of 1,730 publications. After screening the titles and abstracts of all the publications on MERS-CoV, a total of 1,587 publications were included in the final analysis as shown in Figure 3. The data were downloaded both in comma-separated values and plain-text format.

### Data extraction

A number of attributes were extracted, including publication title, year of publication, authors name, journals name, keywords, institution, country of origin, and citations count. The impact factor (IF) of the journals was obtained from the Incites Journal Citation Reports 2020, released by Clarivate Analytics on June 30, 2021.

### Data analysis

The obtained data were exported into HistCite^TM^ software, version 12.3.17; VOSviewer software, version 1.6.17; and Bibliometrix: An R-tool, version 3.2.1 to perform the prerequisite analysis. The citations count was calculated using HistCite^TM^ software (22). Both the local citations score (LCS) and the global citations score (GCS) were calculated. LCS means how many times a paper has been cited by other papers included in the sample (documents or publications analyzed in the current study), while GCS means a paper has been cited by all the included papers in the WoSCC database (23).

Furthermore, the obtained data were plotted for co-authorship authors' network visualization and co-authorship countries or regions overlay visualization mapping using VOSviewer software (24). In addition, the data were imported into RStudio (Bibliometrix package) to analyze inter-countries or regions collaboration, keywords analysis, and trend topics in MERS-CoV research over the years. The calculated values were presented in number/frequency (*n*) and percentage (%).

### Ethics statement

In the current study, no animal or human subjects were recruited directly. Therefore, no ethical consideration was required.

## Results

In this study, a total of 1,587 documents or publications were included in the final analysis. These documents were published in 499 journals, authored by 6,506 authors (4.1 authors per document, 7.92 co-authors per document) from 88 countries. The authors collaboration index was recorded 4.36, as shown in Table 1.

**Table 1:** Main information about the included and analyzed publications on MERS-CoV between 2012 and 2021.

**Description**	**Results**
**Main information**	
Time-Span	2012–2021
Journals	499
Documents or publications	1,587
Institutions (affiliations)	1,627
Countries or regions	88
Average years from publication	4.89
Average citations per document	44.2
Average citations per year per document	7.134
Local citations score	14,139
Global citations score	70,143
References	22,459
**Document types**	
Research article	1,143
Review	183
Editorial material	130
Letter	108
Proceedings paper	23
**Document contents**	
KeyWords Plus	1,660
Author's keywords	2,062
**Authors**	
Authors	6,506
Author appearances	12,572
Authors of single-authored documents	90
Authors of multi-authored documents	6,416
**Authors collaboration**	
Single-Authored documents	117
Documents per author	0.244
Authors per document	4.1
Co-Authors per document	7.92
Collaboration index	4.36

### Most cited publications and frequent years

The included publications received a total of 14,139 LCS and 70,143 GCS (Table 1). The most cited publication was “Isolation of a Novel Coronavirus from a Man with Pneumonia in Saudi Arabia,” published in New England Journal of Medicine in 2012, received 2,994 citations (272.18 citations per year) as shown in Table 2. The most frequent years of publication were 2016 (*n* = 253), and 2015 (*n* = 206), while the most cited years were 2014 (11,517 citations), and 2013 (11,173 citations), as presented in Table 3.

**Table 2:** Top 10 most-cited publications on MERS-CoV according to GCS at the time of search.

**Ranking**	**Publication title**	**LCS**	**LCS per year**	**GCS**	**GCS per year**	**References**
1	Isolation of a Novel Coronavirus from a Man with Pneumonia in Saudi Arabia	783	71.18	2,994	272.18	(1)
2	Comparative therapeutic efficacy of remdesivir and combination lopinavir, ritonavir, and interferon beta against MERS-CoV	0	0.00	873	291.00	(25)
3	Epidemiological, demographic, and clinical characteristics of 47 cases of Middle East respiratory syndrome coronavirus disease from Saudi Arabia: a descriptive study	295	29.50	841	84.10	(26)
4	Hospital outbreak of Middle East respiratory syndrome coronavirus	418	41.80	787	78.70	(27)
5	Middle East Respiratory Syndrome Coronavirus (MERS-CoV): Announcement of the Coronavirus Study Group	0	0.00	676	67.60	(5)
6	Corticosteroid therapy for critically ill patients with Middle East respiratory syndrome	18	3.60	616	123.20	(28)
7	Middle East respiratory syndrome	176	22.00	589	73.63	(29)
8	Middle East respiratory syndrome coronavirus: another zoonotic betacoronavirus causing SARS-like disease	87	10.88	481	60.13	(30)
9	Middle East respiratory syndrome coronavirus neutralizing serum antibodies in dromedary camels: a comparative serological study	233	23.30	467	46.70	(31)
10	Prophylactic and therapeutic remdesivir (GS-5734) treatment in the rhesus macaque model of MERS-CoV infection	0	0.00	437	145.67	(32)

**Table 3:** Year of publications.

**Publications year**	**Number of published papers**	**Percentage**	**LCS**	**GCS**
2012	8	0.5	823	3,411
2013	100	6.3	3,700	11,173
2014	153	9.6	3,116	11,517
2015	206	13.0	2,622	9,513
2016	253	15.9	2,022	10,380
2017	182	11.5	847	6,582
2018	151	9.5	541	5,734
2019	177	11.2	313	4,556
2020	190	12.0	135	6,474
2021	167	10.6	20	803

### Publication types, leading authors, and journals

The publication types were research article (*n* = 1,143), review (*n* = 183), editorial material (*n* = 130), letter (*n* = 108), and proceedings paper (*n* = 23), as presented in Table 4. Based on the number of publications, the most active authors were Memish ZA (*n* = 94), Al-Tawfiq (*n* = 71), Drosten C (*n* = 45), Haagmans BL (*n* = 42), and Gerber SI (*n* = 38) as shown in Table 5. The Emerging Infectious Diseases was the leading journal (*n* = 80), followed by Journal of Virology (*n* = 65), and Journal of Infection and Public Health (*n* = 44), as shown in Table 6.

**Table 4:** Types of publication.

**Publication types**	**Number of published papers**	**Percentage**	**LCS**	**GCS**
Research article	1,143	72.0	12,373	58,065
Review	183	11.5	835	7,808
Editorial material	130	8.2	430	1,960
Letter	108	6.8	469	1,965
Proceedings paper	23	1.4	32	345

**Table 5:** Top 10 most prolific authors based on number of publications.

**Ranking**	**Author**	**Affiliation**	**Number of published papers**	**Percentage**	**LCS**	**LCS per year**	**GCS**	**GCS per year**
1	Ziad A. Memish	Research Center, King Saud Medical City, Ministry of Health, Riyadh, Saudi Arabia	94	5.9	2,628	303.90	8,630	1,148.95
2	Jaffar A. Al-Tawfiq	Infectious Disease Unit, Specialty Internal Medicine, Johns Hopkins Aramco Healthcare, Dhahran, Saudi Arabia	71	4.5	1,453	162.94	4,861	650.46
3	Christian Drosten	Institute of Virology, Charité—Universitätsmedizin Berlin, Helmut-Ruska-Haus Charitéplatz 1, 10117 Berlin, Germany	45	2.8	1,451	166.08	4,745	567.60
4	Bart L. Haagmans	Department of Viroscience, Erasmus Medical Center, Rotterdam, The Netherlands	42	2.6	1,109	134.34	2,985	397.60
5	Susan I. Gerber	Centers for Disease Control and Prevention, Atlanta, Georgia, USA	38	2.4	677	95.59	1,976	299.04
6	Shibo Jiang	School of Basic Medical Sciences and Shanghai Public Health Clinical Center, Fudan University, Shanghai 200032, China	37	2.3	592	72.65	2,436	331.60
7	Stanley Perlman	Department of Microbiology and Immunology, and Department of Pediatrics, University of Iowa, Iowa City, IA, USA	35	2.2	586	78.76	3,315	489.17
8	^*^Yaseen M. Arabi	King Saud Bin Abdulaziz University for Health Sciences Riyadh, Saudi Arabia	33	2.1	368	56.24	2,671	500.43
8	^*^Jun-Young Lee	Korea Research Institute of Chemical Technology, 141 Gajeong-ro, Yuseong-gu, Daejeon 34114, South Korea	33	2.1	255	40.48	1,024	181.61
9	Alimuddin Zumla	Division of Infection and Immunity, University College London, London, United Kingdom	32	2.0	713	92.56	2,226	346.18
10	Malik Peiris	School of Public Health, The University of Hong Kong, No 7 Sassoon Rd, Pokfulam, Hong Kong	30	1.9	333	49.13	984	142.85

* *Both the authors produced equal number of papers*.

**Table 6:** Top 10 most published journals.

**Ranking**	**Journal**	**IF 2020**	**Number of published papers**	**Percentage**	**LCS**	**LCS per year**	**GCS**	**GCS per year**	**Local cited references**
1	Emerging Infectious Diseases	6.883	80	5.0	1,360	182.41	3,357	490.62	566
2	Journal of Virology	5.103	65	4.1	1,353	150.12	5,250	665.32	547
3	Journal of Infection and Public Health	3.718	44	2.8	121	24.00	989	229.70	384
4	Eurosurveillance	6.307	40	2.5	90	10.27	2,125	231.09	135
5	Viruses-Basel	5.048	37	2.3	0	0.00	682	158.62	659
6	International Journal of Infectious Diseases	3.623	33	2.1	543	66.04	1,998	299.07	211
7	Plos One	3.24	31	2.0	0	0.00	1,062	144.26	256
8	Lancet Infectious Diseases	25.071	27	1.7	1,293	146.94	3,303	423.49	184
9	Journal of Infectious Diseases	5.226	26	1.6	488	63.84	1,909	261.27	266
10	^*^Clinical Infectious Diseases	9.079	25	1.6	373	56.09	1,523	242.62	186
10	^*^Scientific Reports	4.38	25	1.6	0	0.00	677	120.52	264

* *Both the journals published equal number of papers*.

### Most studied research areas, funding agencies, and most frequent publishers

The extensively studied research areas were Infectious Disease (*n* = 513), Public Environmental Occupational Health (*n* = 246), Immunology (*n* = 228), Virology (*n* = 224), and Microbiology (*n* = 191). The most number of publications was funded by United States Department of Health and Human Services (HHS) (*n* = 256), followed by National Institutes of Health (NIH), United States of America (USA) (*n* = 230), National Institute of Allergy Infectious Diseases (NIAID), USA (*n* = 178), European Commission (*n* = 86), and National Natural Science Foundation of China (NSFC) (*n* = 72). The most frequent publishers were Elsevier (*n* = 375), Springer Nature (*n* = 159), American Society for Microbiology (*n* = 105), Wiley (*n* = 76), and Oxford University Press (*n* = 72).

### Most active institutions and countries

The most active institution was Ministry of Health, Saudi Arabia (*n* = 135), followed by University of Hong Kong, Hong Kong (*n* = 94), King Saud University, Saudi Arabia (*n* = 83), King Saud Bin Abdulaziz University for Health Sciences, Saudi Arabia (*n* = 76), and Alfaisal University, Saudi Arabia (*n* = 72), as presented in Table 7. The largest number of publications was produced by the USA (*n* = 520), followed by Saudi Arabia (*n* = 432), China (*n* = 301), South Korea (*n* = 241), and the United Kingdom (*n* = 121), as presented in Table 8.

**Table 7:** Top 10 leading institutions in MERS-CoV research.

**Ranking**	**Institution**	**Country/Region**	**Number of published papers**	**Percentage**	**LCS**	**GCS**
1	Ministry of Health	Saudi Arabia	135	8.5	2,820	9,087
2	University of Hong Kong	Hong Kong	94	5.9	1,312	6,556
3	King Saud University	Saudi Arabia	83	5.2	565	4,032
4	King Saud Bin Abdulaziz University for Health Sciences	Saudi Arabia	76	4.8	505	3,600
5	Alfaisal University	Saudi Arabia	72	4.5	892	4,959
6	Seoul National University	South Korea	61	3.8	357	2,054
7	National Institute of Allergy and Infectious Diseases	USA	60	3.8	416	4,911
8	Johns Hopkins Aramco Healthcare	Saudi Arabia	57	3.6	494	2,250
9	Indiana University School of Medicine	USA	56	3.5	617	2,734
10	King Faisal Specialist Hospital and Research Center	Saudi Arabia	55	3.5	1,107	4,572

**Table 8:** Top 10 highly productive countries in MERS-CoV research.

**Ranking**	**Country**	**Number of published papers**	**Percentage**	**LCS**	**GCS**
1	USA	520	32.8	6,025	30,523
2	Saudi Arabia	432	27.2	5,139	22,793
3	China	301	19.0	3,001	16,108
4	South Korea	241	15.2	991	6,727
5	United Kingdom	121	7.6	2,709	10,638
6	Egypt	102	6.4	538	4,198
7	Germany	88	5.5	1,865	7,015
8	Netherlands	68	4.3	2,099	8,421
9	Canada	65	4.1	901	5,454
10	France	53	3.3	822	2,971

### Co-authorship authors network visualization

**Figure 4:**
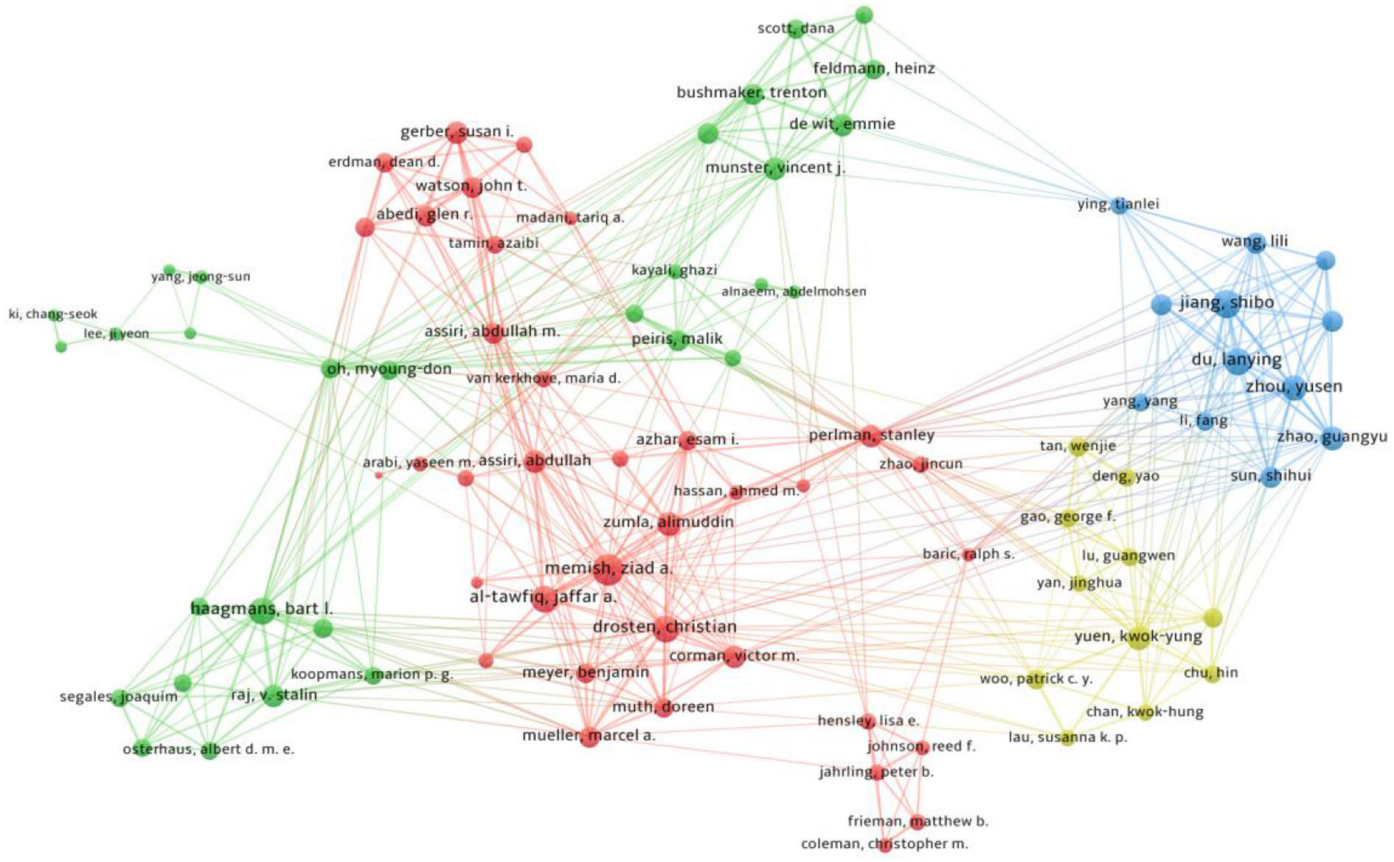
A co-authorship author network visualization map based on total link strength. The thicker the line among the authors, the stronger the collaboration, while the bigger the node, the greatest the contribution (publications).

The minimum number of publications of an author was selected at 10. The minimum cluster size was fixed at 10. Of the total involved authors, only 89 authors met the threshold and were plotted (Figure 4). The most frequent and active authors were plotted into four clusters; Cluster 1 (red color, 36 authors), Cluster 2 (green color, 30 authors), Cluster 3 (blue color, 12 authors), and Cluster 4 (yellow color, 11 authors). As shown in Figure 4, Zaid A. Memish had the strongest collaboration with Jaffar A. Al-Tawfiq; both the authors are from Saudi Arabia.

### Co-authorship countries or regions overlay visualization and countries collaboration

**Figure 5:**
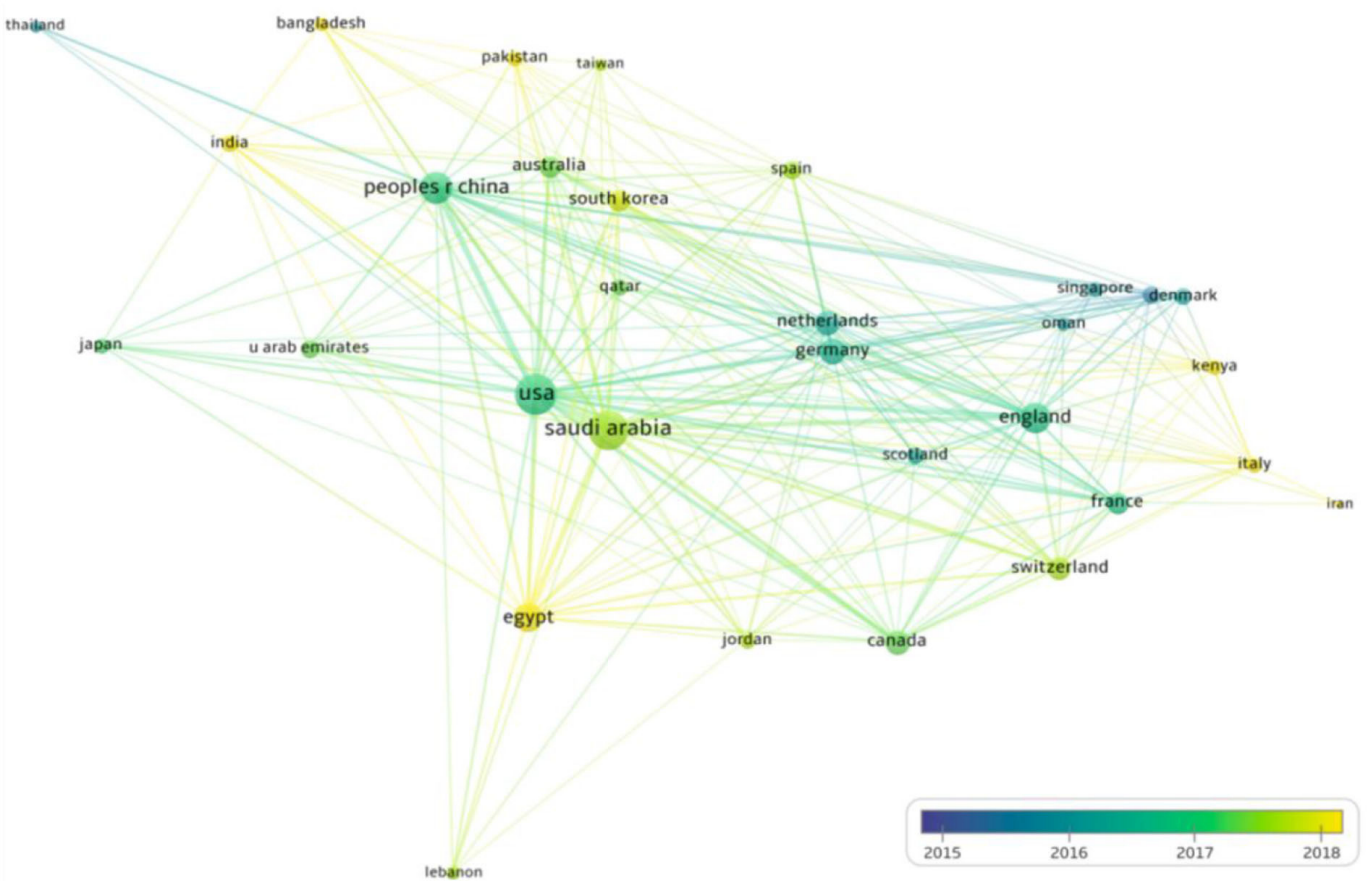
Co-authorship countries or regions overlay visualization over time (years). The thicker the line between two countries or regions, the stronger the collaboration, while the bigger the node, the higher the contribution (publications).

As shown in Figure 5, the countries or regions participated in MERS-CoV-related research over the years were plotted for overlay visualization mapping. The minimum number of documents of a country or region was selected at 10. Of the total countries or regions, only 31 were plotted. The scale was selected as follows: weight (total link strength), scores (average publications per year). The top countries with the highest total link strength were the USA, Saudi Arabia, China, England, and Egypt, 582, 516, 276, 238, and 203, respectively. Interestingly, in recent years, the most active countries in MERS-CoV have been Egypt and South Korea. However, many other developing countries are also participating as described in Figure 5.

**Figure 6:**
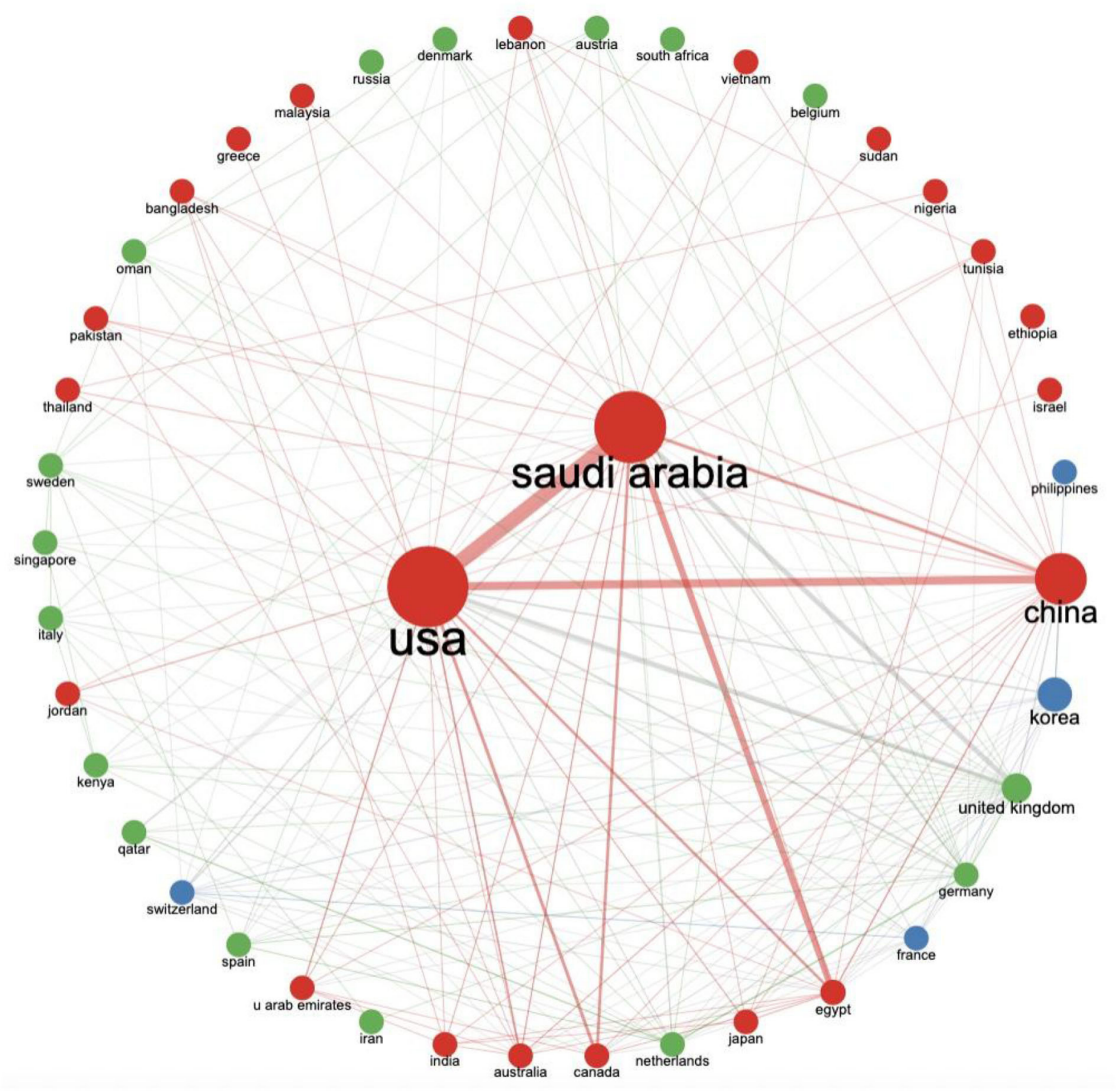
Inter-countries or regions collaboration. The thicker the line between two countries or regions, the stronger the collaboration, while the bigger the node, the higher the contribution (publications).

The obtained data were also plotted for the inter-countries or regions collaboration in MERS-CoV research. As shown in Figure 6, Saudi Arabia had the strongest collaboration with the USA, while the USA had the strongest collaboration with China.

### Keywords analysis and trend topics

**Figure 7:**
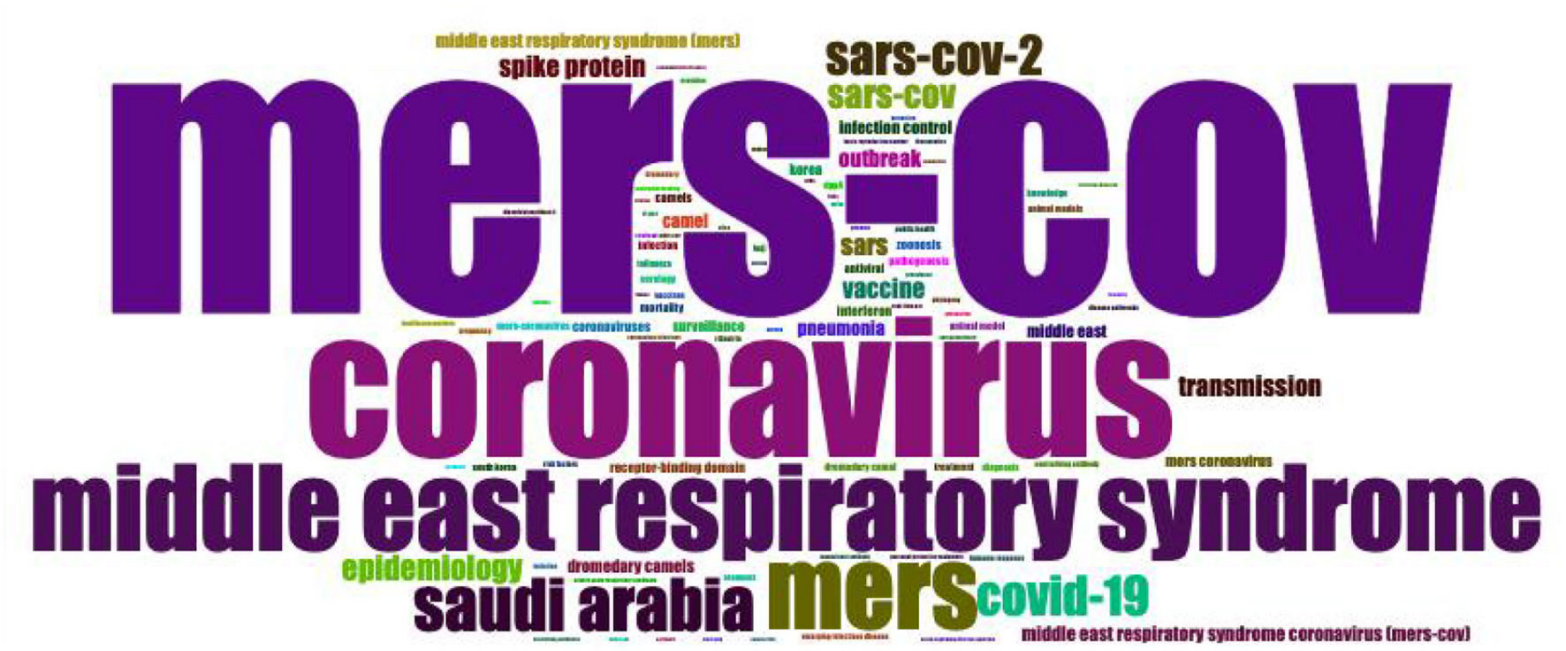
A WordCloud map of frequently used top 100 author's keywords.

The keyword and trend topics analyses were performed using the Bibliometrix package. As shown in Figure 7, the most frequently used author's keywords other than search keywords were Saudi Arabia, SARS-CoV-2, epidemiology, spike protein, transmission, vaccine, outbreak, pneumonia, camel(s), and infection control.

**Figure 8:**
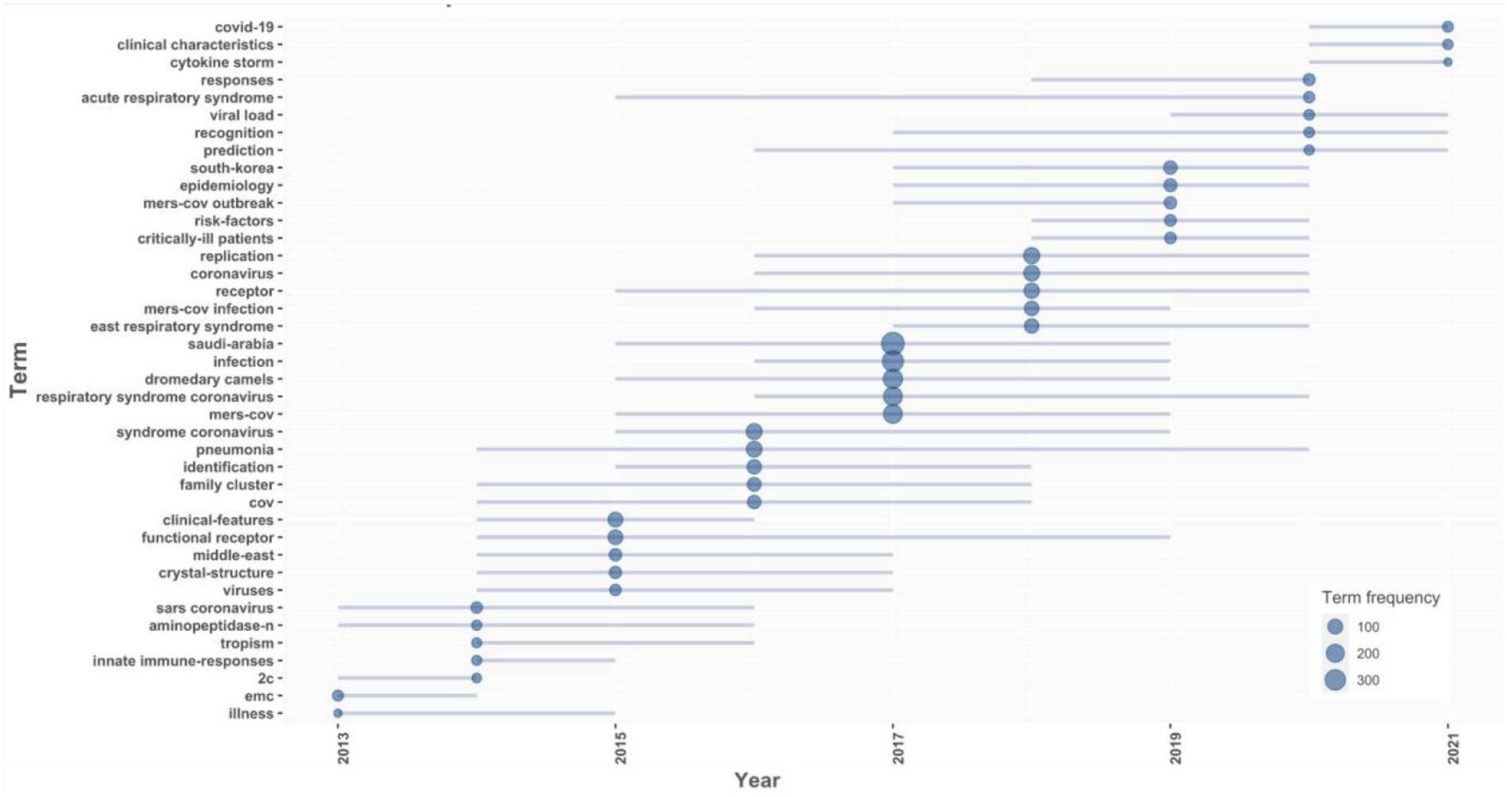
Trend topics in MERS-CoV over the years (2013–2021).

The most studied trend topics over the years were mainly related to Saudi Arabia, MERS-CoV, infection, dromedary camel, replication, coronavirus, pneumonia, receptor, clinical feature, and identifications, as shown in Figure 8. However, COVID-19, clinical characteristics, and cytokine storm were the most studied trend topics in 2021.

## Discussion

This study highlights the global research trends, hotspot research areas, leading authors, journals, institutions, and countries, and citations count and collaboration linkage over the past 10 years in MERS-CoV research.

In this study, a rapid increase has been observed in publications from 2012 (*n* = 8) to 2016 (*n* = 253). While after onward 2016, more than 150 publications have been published in each year. However, the most frequent publication year was 2016, while the most cited year was 2014. Furthermore, the citations of the top 10 publications range from 437 to 2,994 times. The most cited publication was “Isolation of a Novel Coronavirus from a Man with Pneumonia in SaudiArabia” cited 2,994 times (272.18 average citations per year) (1). This was the first publication providing the initial and essential information on MERS-CoV in a 60-year-old patient. The second most cited publication was “Comparative therapeutic efficacy of remdesivir and combination lopinavir, ritonavir, and interferonbeta against MERS-CoV” cited 873 times (291 average citations per year) (25). This paper discusses different therapeutics approaches to treat MERS-CoV infection. All the top 10 most cited publications were published in high-quality and prestigious journals. Of these, the New England Journal of Medicine published two papers, having the IF 91.253 in 2020 (89.676 5-year IF). Furthermore, in total, the most frequently published journal was “Emerging Infectious Diseases” IF = 6.883 in 2020 (7.463 5-year IF). The IF of the top 10 most frequently published journals ranges from 3.24 (Plos One) to 25.071 (Lancet Infectious Diseases) in 2020. The above statistics show that the authors more likely to target the relevant and high-IF journals.

In this study, the leading institution was Ministry of Health, Saudi Arabia, and the second most active country was Saudi Arabia, which might be due to the fact that the first case of MERS-CoV (1) and many outbreaks of MERS-CoV have been reported in Saudi Arabia (33–38). In this study, the USA was the most active and highly contributing country in MERS-CoV global research. The findings of the current study are in line with previously published bibliometric studies conducted in different research fields (39–47). The possible explanation for this is that the USA allocated a considerable budget to research, science, and technology, and strongly collaborated with other developed countries (23).

## Limitations

This study has several limitations: Firstly, the data used in this study were retrieved from a single database. The use of other databases, such as Google Scholar, PubMed, and Scopus, may alter the publications number and citations frequency. Secondly, the search was limited to only English language and document types. Thirdly, the searching keywords were restricted to the title field.

## Conclusion

The current study provides a comprehensive snapshot of 10 years of MERS-CoV research. The findings can be useful for future studies and helpful for researchers, academicians, and policy makers. The Ministry of Health, Saudi Arabia was the most active institution in MERS-CoV research. The highly contributing countries were the USA and Saudi Arabia. However, Saudi Arabia had the strongest collaboration with the USA, while the USA had the strongest collaboration with China. The most frequently used author's keywords other than search keywords were Saudi Arabia, SARS-CoV-2, epidemiology, spike protein, transmission, vaccine, outbreak, pneumonia, camel(s), and infection control. In 2021, the most frequently trend topics in MERS-CoV-related research were COVID-19, clinical characteristics, and cytokine storm.

## Data availability statement

The original contributions presented in the study are included in the article/supplementary material, further inquiries can be directed to the corresponding author.

## Author contributions

TA: conceptualization, study design and methods, data curation and extraction, software and analysis, and writing—original draft.

## Conflict of interest

The author declares that the research was conducted in the absence of any commercial or financial relationships that could be construed as a potential conflict of interest.

## Publisher's note

All claims expressed in this article are solely those of the authors and do not necessarily represent those of their affiliated organizations, or those of the publisher, the editors and the reviewers. Any product that may be evaluated in this article, or claim that may be made by its manufacturer, is not guaranteed or endorsed by the publisher.

## Abbreviations

GCS: Global citations score
IF: Impact Factor
HHS: United States Department of Health and Human Services
LCS: Local citations score
MERS-CoV: Middle East respiratory syndrome coronavirus
NIH: National Institutes of Health
NIAID: National Institute of Allergy Infectious Diseases
NSFC: National Natural Science Foundation of China
SCI-Expanded: Science Citation Index Expanded
USA: United States of America
WHO: World Health Organization.
